# Supplementary material for: Fully Automated Macro- and Microfluidic Production of [68Ga]Ga-Citrate on mAIO® and iMiDEVTM Modules
Source: Molecules. 2022 Feb 1;27(3):994. doi: 10.3390/molecules27030994 (PMC8838513; doi:10.3390/molecules27030994)
Supplement: Supplementary file 1 [file molecules-27-00994-s001.zip › molecules-1540852-supplementary.pdf]

Supplementary Materials:

Fully Automated Macro- and Microfluidic Production of [^{68}Ga]Ga-citrate on mAIO® and iMiDEV™ modules

Olga Ovdiichuk [1]*, Emilie Roeder [1], Sébastien Billotte[1], Nicolas Veran [2], Charlotte Collet [1,3]*

1 Nancyclotep, Molecular Imaging platform, F-54500 Vandoeuvre-les-nancy, France
2 CHRU-Nancy, Service de Medecine Nucléaire, Pôle Pharmacie, F-54000 Nancy, France
3 Université de Lorraine, Inserm, IADI, F-54000 Nancy, France

Corresponding authors E-mail addresses:
Charlotte Collet Email: charlotte.collet@univ-lorraine.fr
Olga Ovdiichuk Email: o.ovdiichuk@nancyclotep.com

Table of Contents

Figure S1. Radioactivity curve of trap-release process observed during radiosynthesis of [^{68}Ga]Ga-citrate on mAIO®.

Figure S2. Schematic representation of the steps used for [^{68}Ga]Ga-citrate synthesis in R1 chamber: a) activity reception ; b) washing; c) elution and delivery of [^{68}Ga]Ga-citrate.

Figure S3. Radioactivity curve of trap-release process observed during radiosynthesis of [^{68}Ga]Ga-citrate on R1 chamber filled with PS-H+ on iMiDEV™.

Figure S4. Comparison of radiochromatograms obtained using different systems described in the literature for [^{68}Ga]Ga-citrate radiochemical purity determination.

Figure S5. Representative radio-TLC of [^{68}Ga]Ga-citrate in serum 1h post-incubation at 37 °C.

Figure S6. Schematic representation of the steps used for [^{68}Ga]Ga-citrate synthesis in R3 chamber: a) activity reception ; b) washing; c) elution and delivery of [^{68}Ga]Ga-citrate.

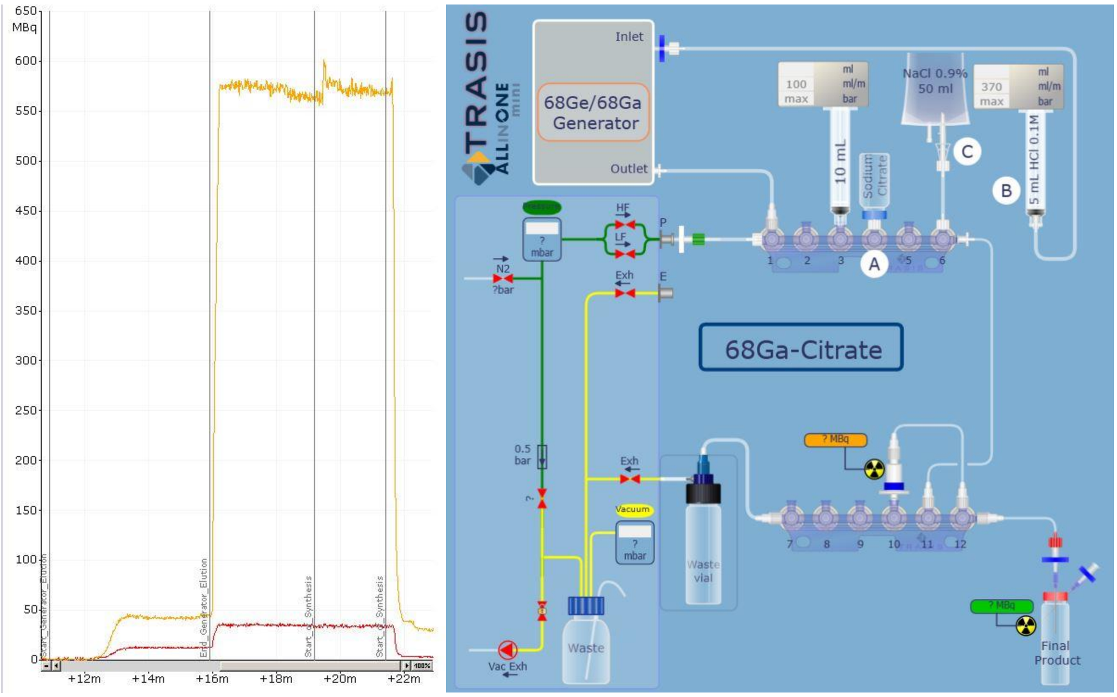

Figure S1. Radioactivity curve of trap-release process observed during radiosynthesis of [^{68}Ga]Ga-citrate on mAIO®. Highlighted in orange – radioactive detector place near the position 10. highlighted in red – radioactive detector placed next to the oven.

a)

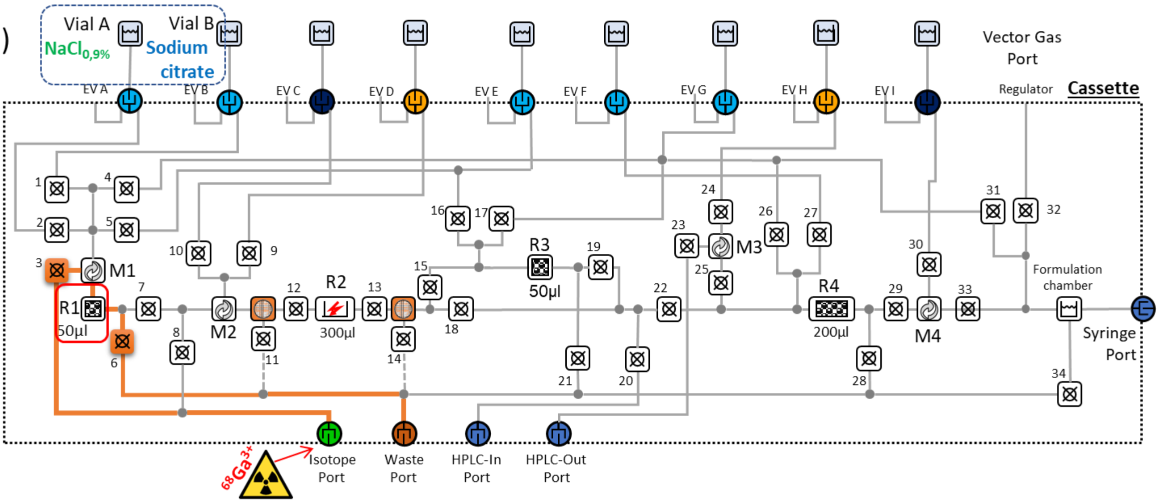

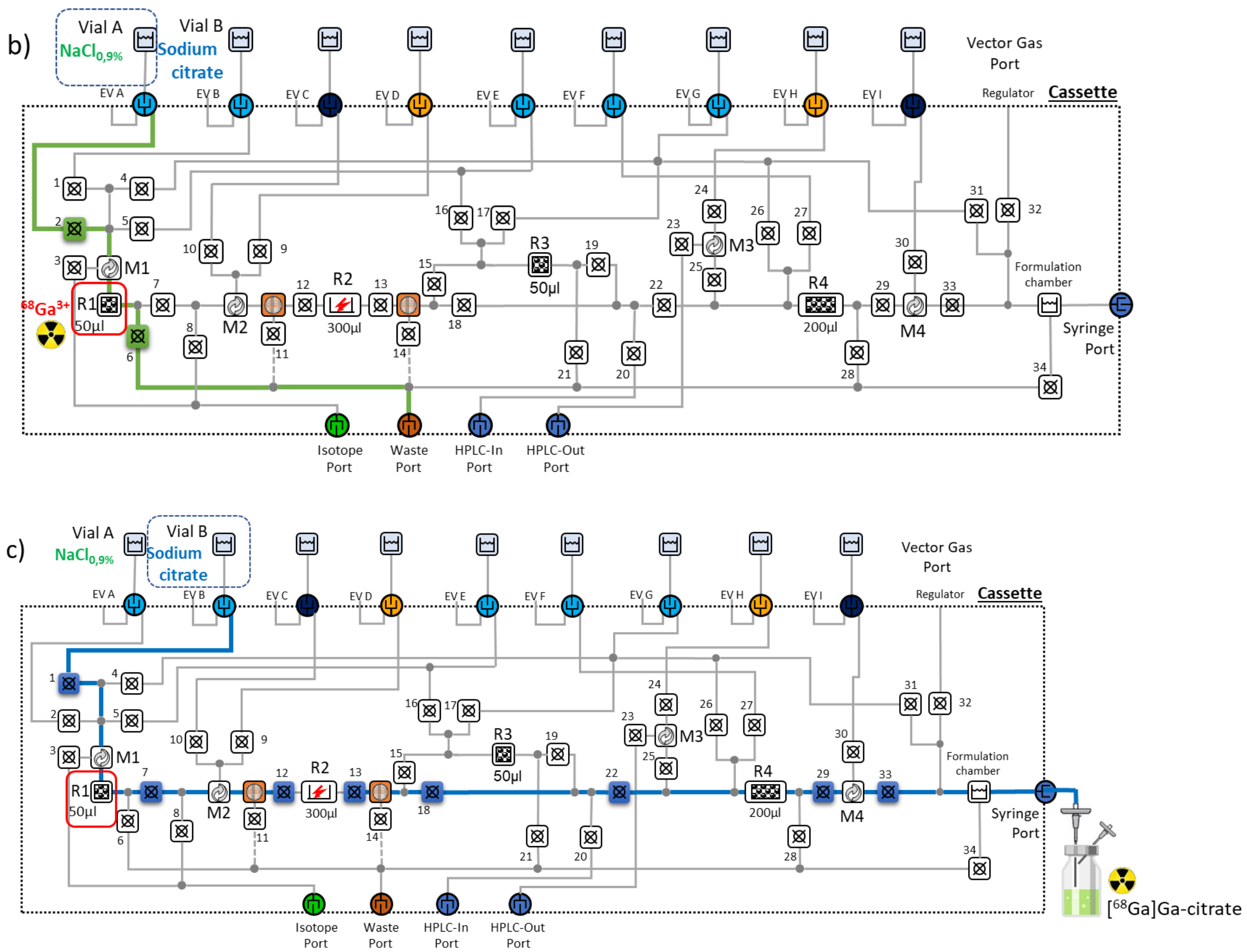

Figure S2. Schematic representation of the steps used for $[^{68}Ga]$Ga-citrate synthesis in R1 chamber: a) activity reception ; b) washing; c) elution and delivery of $[^{68}Ga]$Ga-citrate.

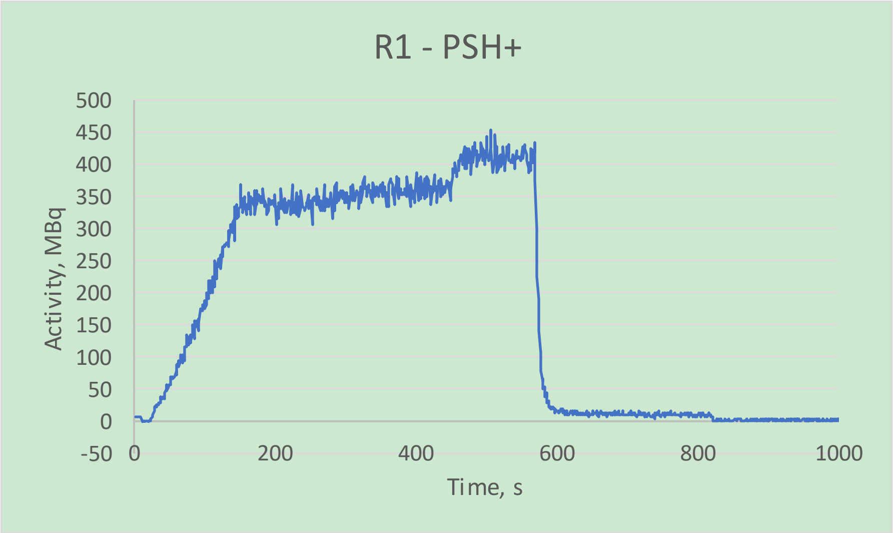

Figure S3. Radioactivity curve of trap-release process observed during radiosynthesis of $[^{68}Ga]$Ga-citrate on R1 chamber filled with PS-H+ on iMiDEV™; Starting activity – 417 MBq.

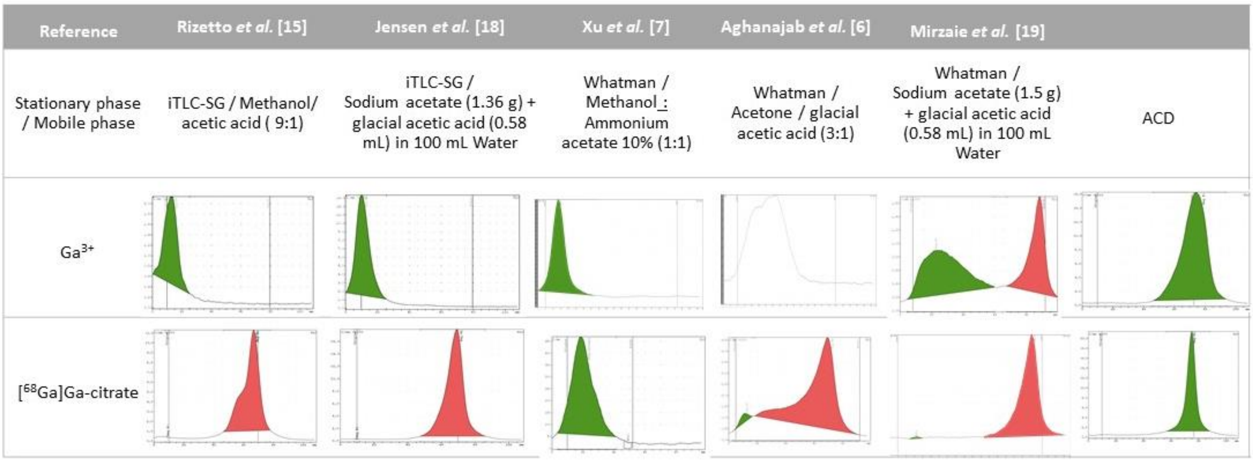

Figure S4. Comparison of radiochromatograms obtained using different systems described in the literature for [^{68}Ga]Ga-citrate radiochemical purity determination.

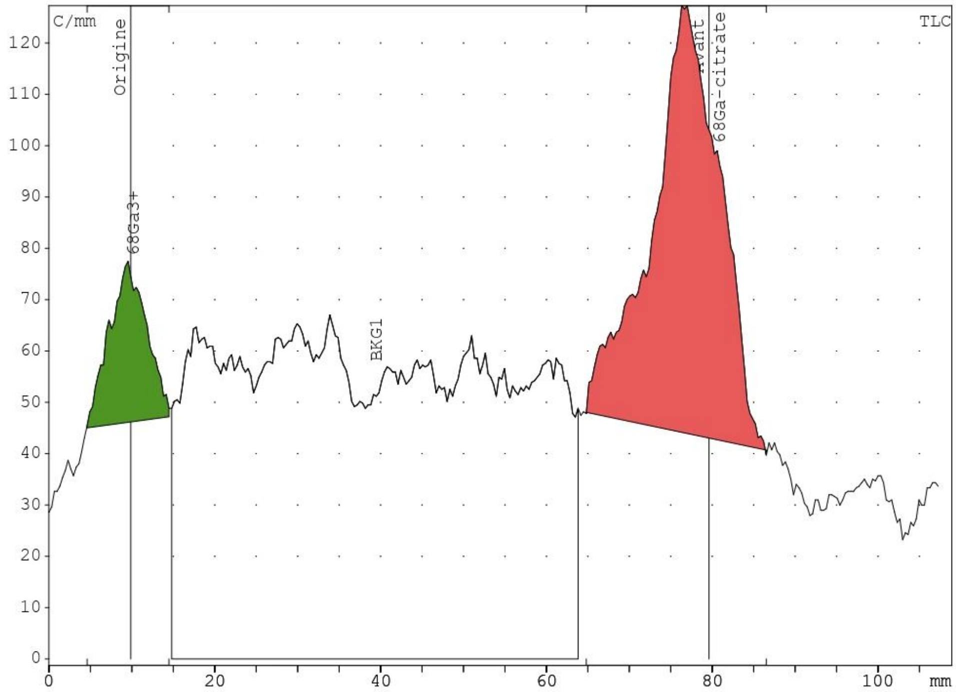

Description de l'échantillon

Etude:	Gallium-68		
Mesure:	Ga1306 serum 10uL 1h, démarré:		02/11/2021 12:32
Méthode:	GALLIUM	Date:	02/11/2021 12:32
Origine:	10 mm	Avant:	80 mm
Temps mesure:	0:05:00 h:m:s	Résolution:	0,33 mm
Haute tension:	890,0 V		
Phase mobile: AcONa 1.5g / AcOH 0.58mL / H2O 100mL ; 10µL			
Détecteur de radioactivité: Elysia-raytest miniGita (BGO)			Numéro de série: 1701198
Version du logiciel: 6.3, service pack 2, build 6415			

Intégration TLC

Substance	R/T mm	R/F	%Total %	Aire Counts	%Aire %
68Ga3+	10	0,000	4,47	174,5714	17,81
68Ga-citrate	76	0,953	20,64	805,5714	82,19
Somme en ROI	-	-	-	980,1429	100,00
Aire générale	-	-	-	3903,5235	-
Aire (total) RF	-	-	-	465,1007	-
BKG1 (CPS)	-	-	-	56,41651	-
Reste (total)	-	-	-	2923,38	74,89

Figure S5. Representative radio-TLC of [^{68}Ga]Ga-citrate in serum 1h post-incubation at 37 °C.

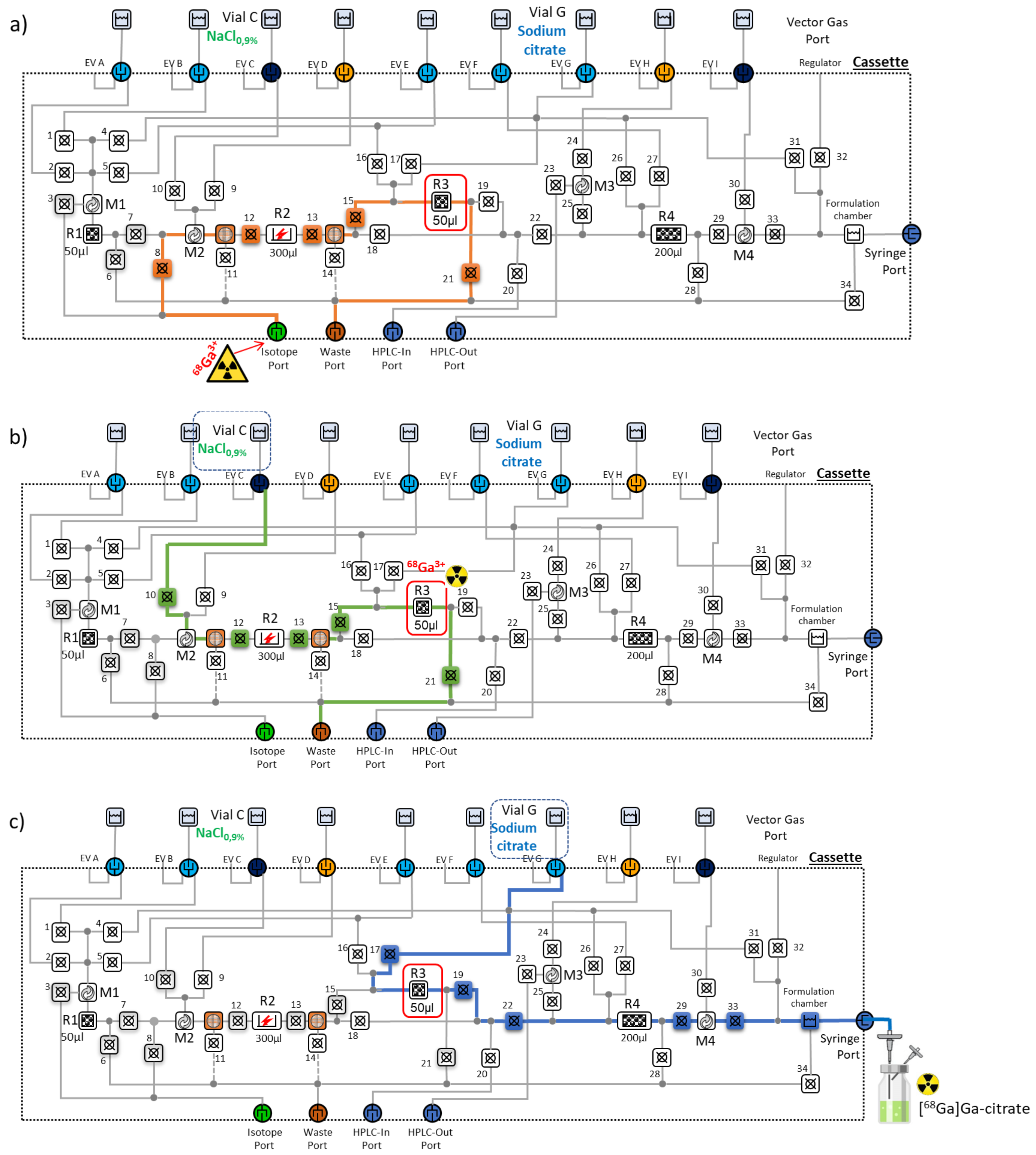

Figure S6. Schematic representation of the steps used for [^{68}Ga]Ga-citrate synthesis in R3 chamber: a) activity reception ; b) washing; c) elution and delivery of [^{68}Ga]Ga-citrate.
